# Supplementary material for: Erubescensoic Acid, a New Polyketide and a Xanthonopyrone SPF-3059-26 from the Culture of the Marine Sponge-Associated Fungus Penicillium erubescens KUFA 0220 and Antibacterial Activity Evaluation of Some of Its Constituents
Source: Molecules. 2019 Jan 8;24(1):208. doi: 10.3390/molecules24010208 (PMC6337093; doi:10.3390/molecules24010208)
Supplement: Supplementary file 1 [file molecules-24-00208-s001.pdf]

Supplementary Materials

Erubescensoic Acid, a New Polyketide and a Xanthonopyrone from the Culture of the Marine Sponge-Associated Fungus *Penicillium erubescens* KUFA 0220 and Antibacterial Activity Evaluation of some of its Constituents

Decha Kumla[1,2], Tida Dethoup[3], Luís Gales[1,4], José A. Pereira[1,2], Joana Freitas -Silva [1, 2], Paulo M. Costa [1,2], Artur M. S. Silva[5], Madalena M. M. Pinto[2,6*], and Anake Kijjoa [1, 2*]

[1] *ICBAS*-Instituto de Ciências Biomédicas Abel Salazar, Universidade do Porto, Rua de Jorge Viterbo Ferreira, 228, 4050-313 Porto, Portugal. E-mail: Decha1987@hotmail.com (D.C.), jpereira@icbas.up.pt (J. A. P.); lgales@ibmc.up.pt (L.G.); pmcosta@icbas.up.pt (P.M.C.).

[2] Interdisciplinary Centre of Marine and Environmental Research (CIIMAR), Terminal de Cruzeiros do Porto de Lexões, Av. General Norton de Matos s/n, 4450-208 Matosinhos, Portugal. E-mail: joanafreitasdasilva@gmail.com.

[3] Department of Plant Pathology, Faculty of Agriculture, Kasetsart University, Bangkok, 10240, Thailand. E-mail: tdethoup@yahoo.com.

[4] Instituto de Biologia Molecular e Celular (i3S-IBMC), Universidade do Porto, Rua de Jorge Viterbo Ferreira, 228, 4050-313 Porto, Portugal.

[5] Departamento de Química & QOPNA, Universidade de Aveiro, 3810-193 Aveiro, Portugal. E-mail: artur.silva@ua.pt.

[6] Laboratório de Química Orgânica, Departamento de Ciências Químicas, Faculdade de Farmácia, Universidade do Porto, Rua de Jorge Viterbo Ferreira, 228, 4050-3 13 Porto, Portugal. E-mail: madalena@ff.up.pt.

*To whom correspondence should be addressed, E-mail: ankijjoa@icbas.up.pt (A.K.), Tel.: +351-220428331; madalena@ff.up.pt.

Figure S1. ^{1}HNMR spectrum of erubescensoic acid (**1**) (DMSO-d_6, 500 MHz).

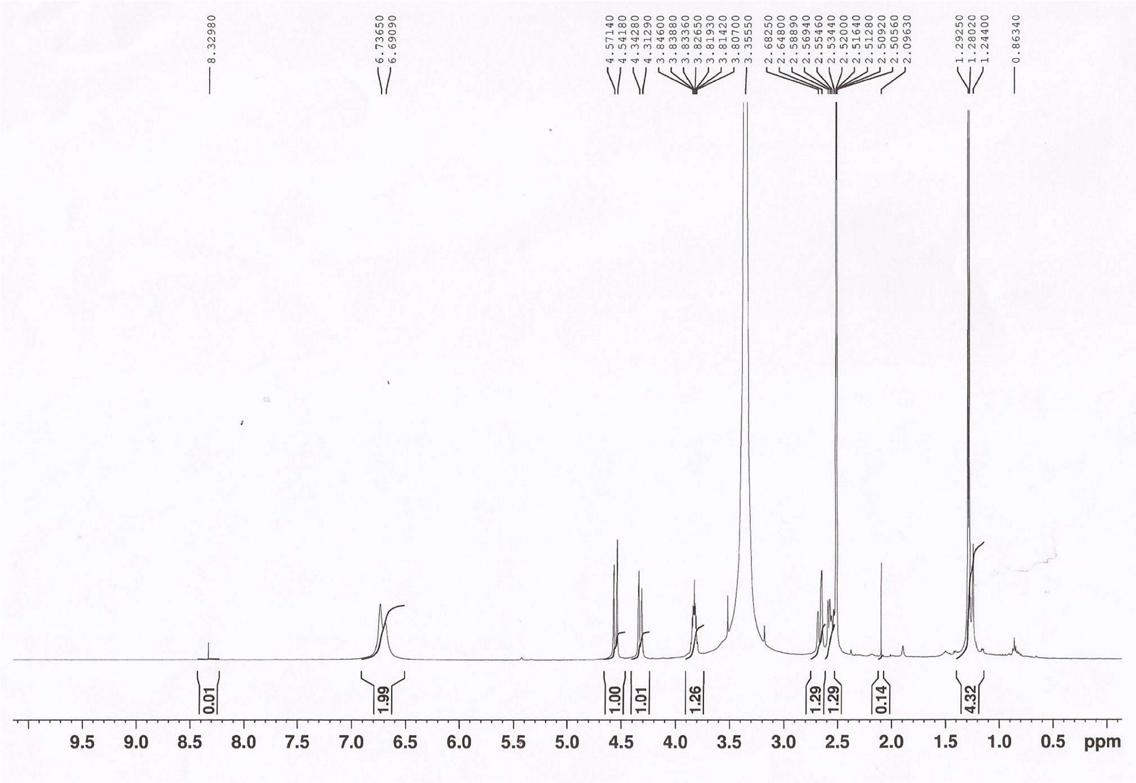

Figure S2. ^{13}C NMR spectrum of erubescensoic acid (**1**) (DMSO-d_6, 125 MHz).

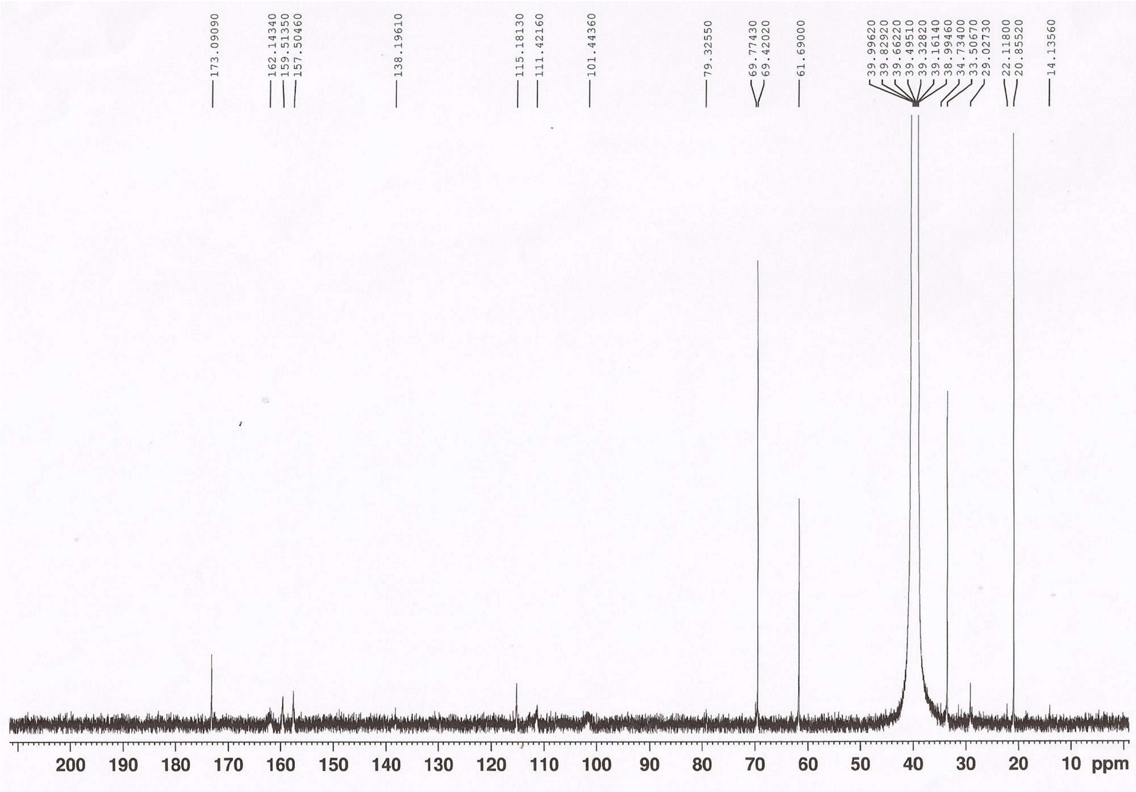

Figure S3. HSQC spectrum of erubescensoic acid (**1**) (DMSO-d$_6$, 500 MHz).

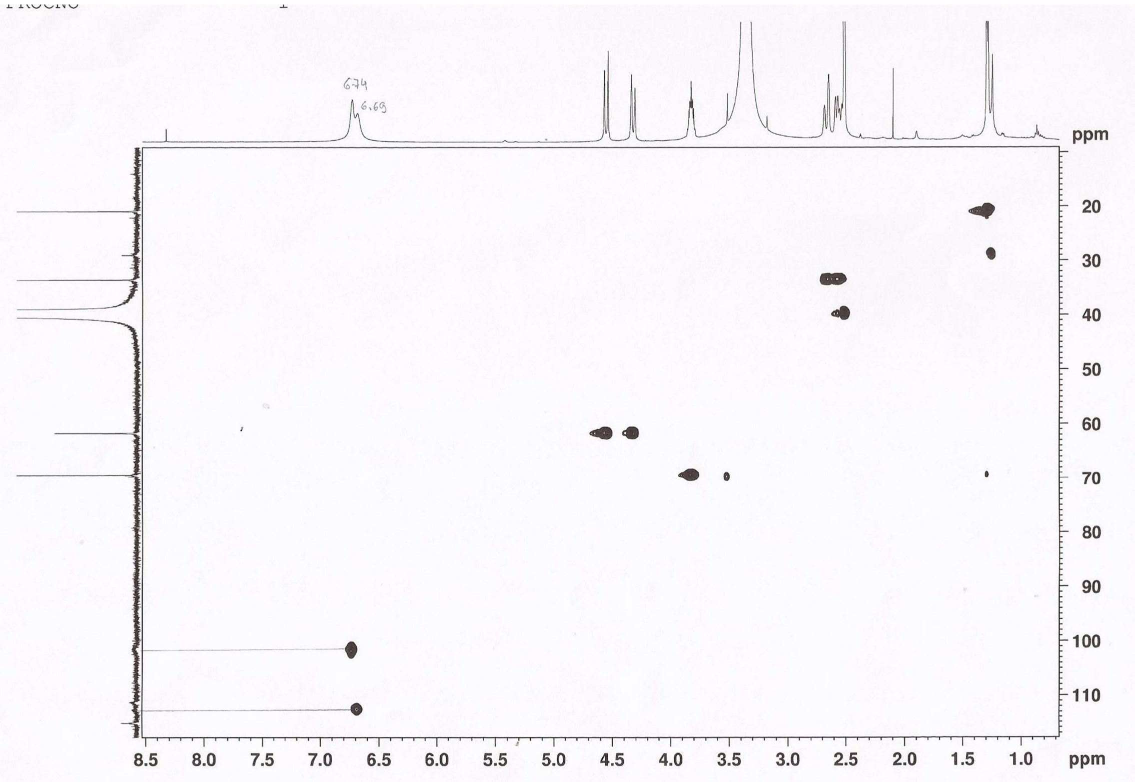

Figure S4. HMBC spectrum of erubescensoic acid (**1**) (DMSO-d$_6$, 500 MHz).

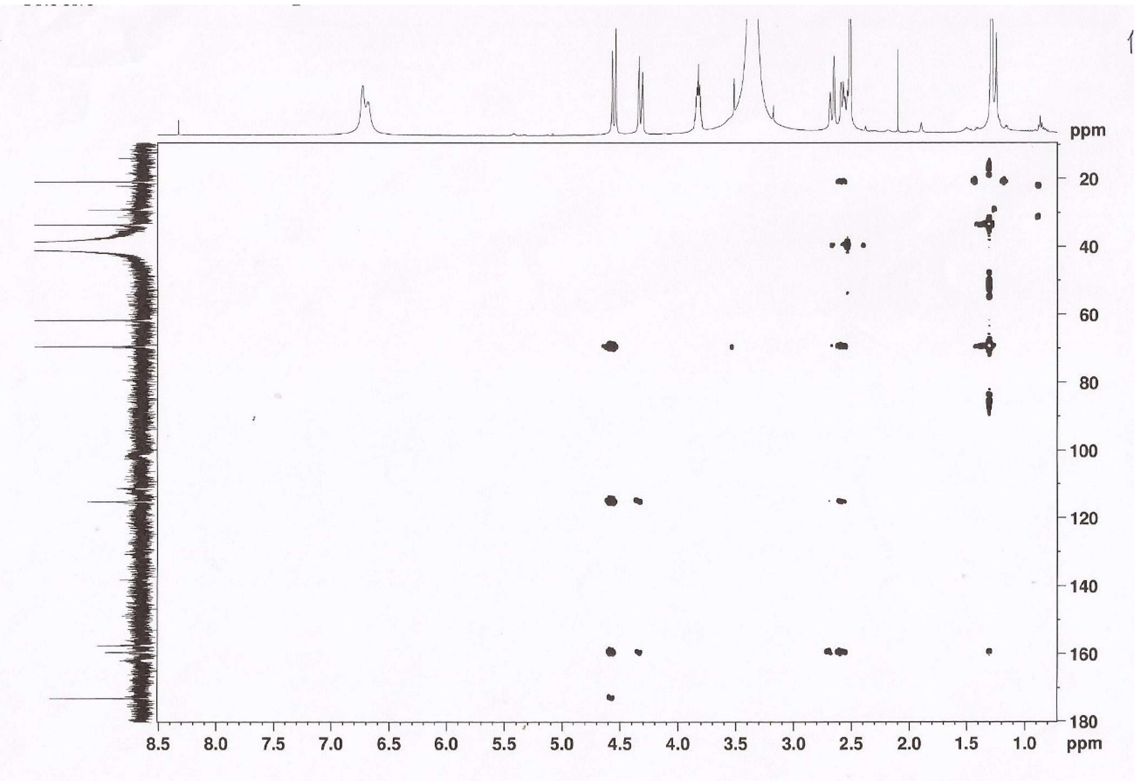

Figure S5. ^{1}H NMR spectrum of SPF-3059-26 (**2**), (DMSO-d_6, 500 MHz).

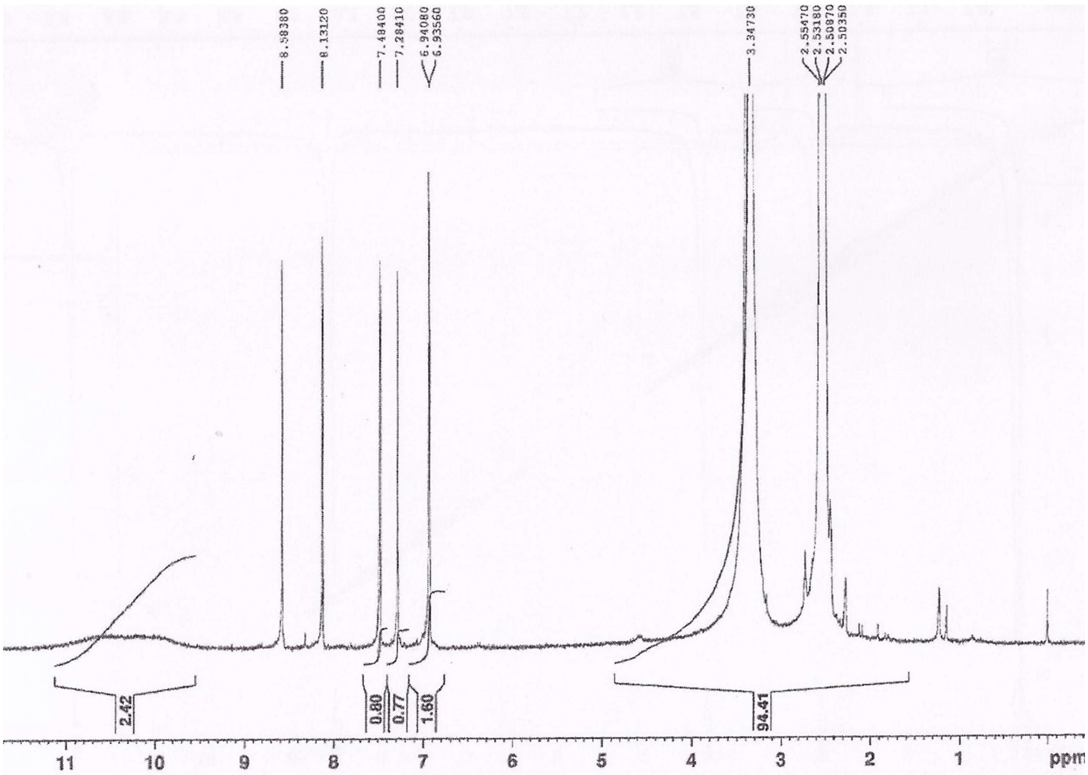

Figure S6. Expansion of the ^{1}H NMR spectrum of SPF-3059-26 (**2**), (DMSO-d_6, 500 MHz).

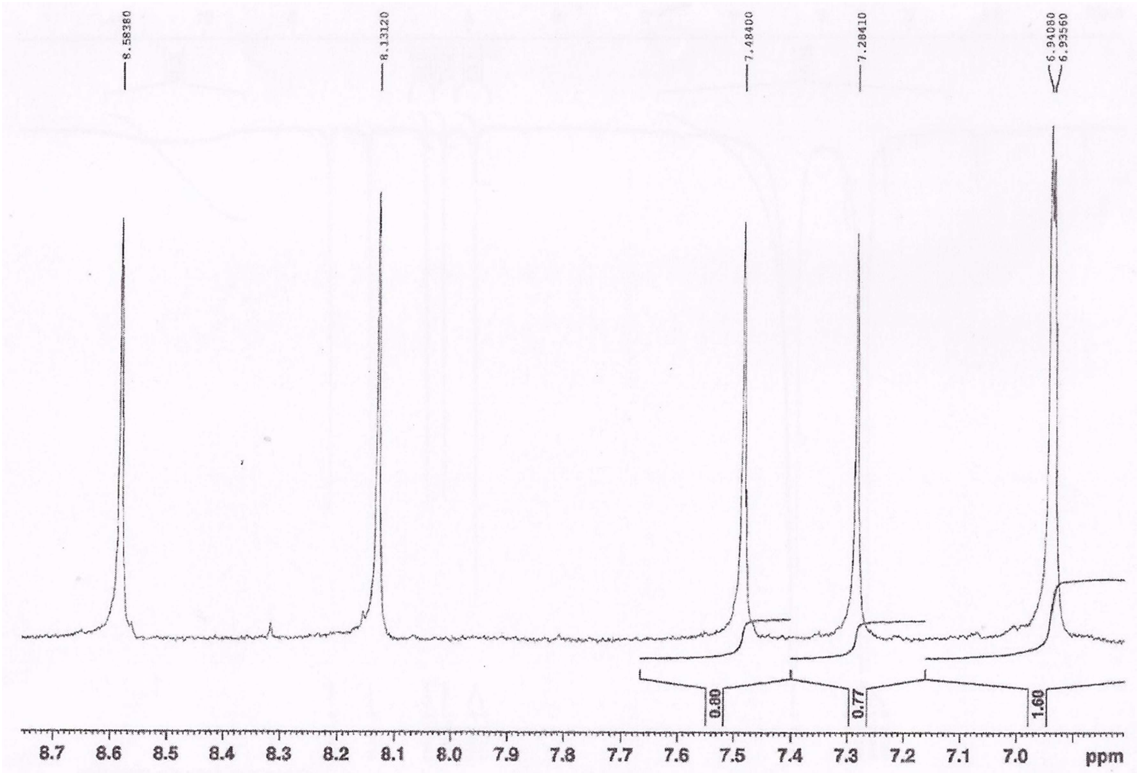

Figure S7. ^{13}C NMR spectrum of SPF-3059-26 (**2**) (DMSO-d$_6$, 125 MHz).

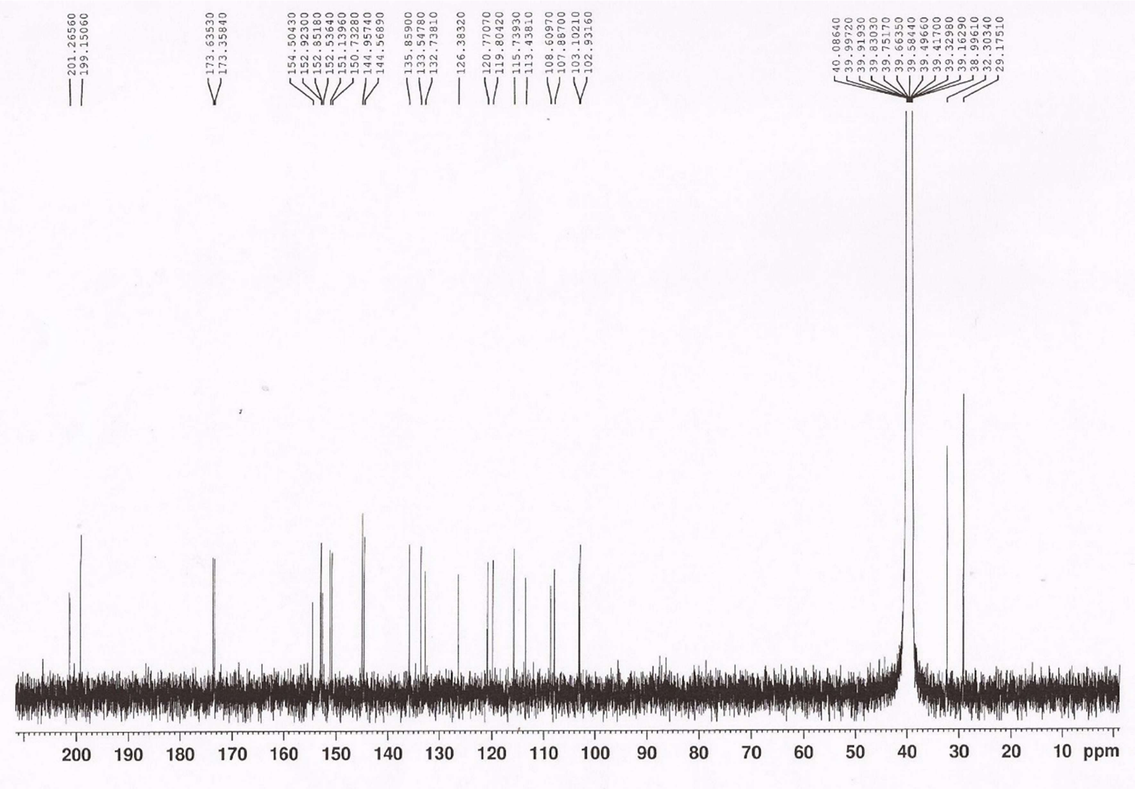

Figure S8. ^{13}C DEPT 135 spectrum of SPF-3059-26 (**2**) (DMSO-d$_6$, 125 MHz).

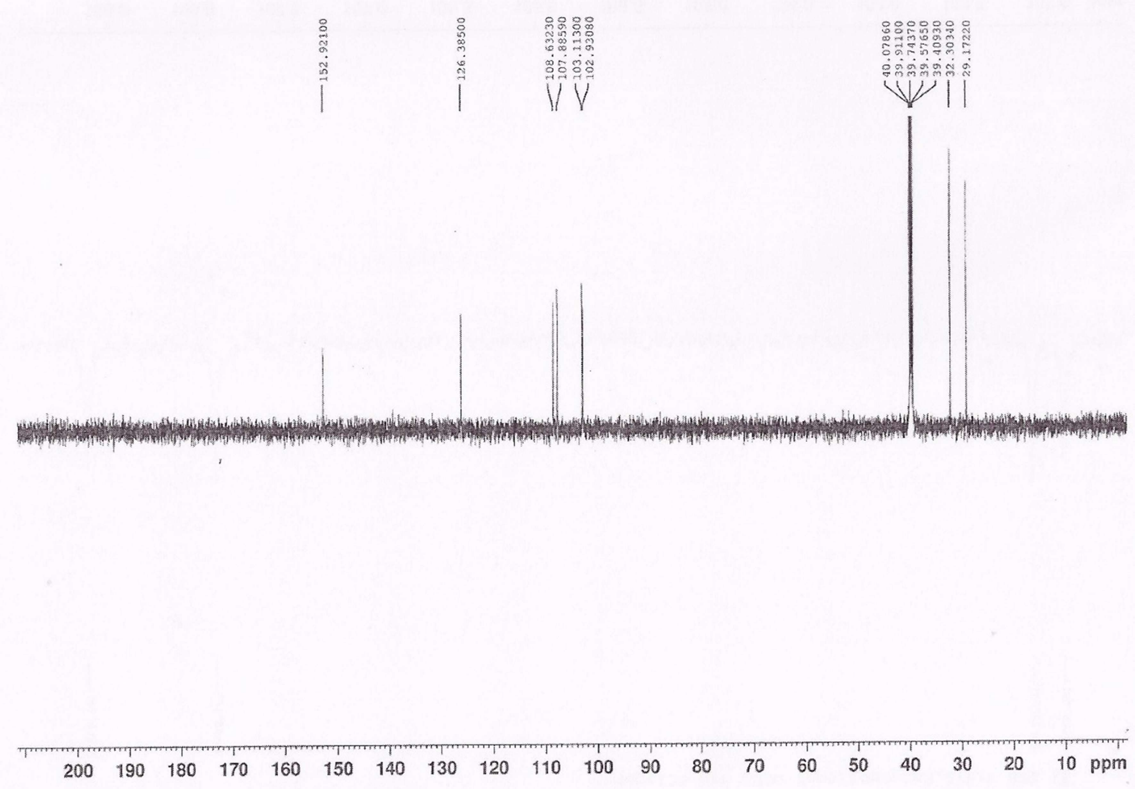

Figure S9. HSQC spectrum of SPF-3059-26 (**2**) (DMSO-d_6, 500 MHz).

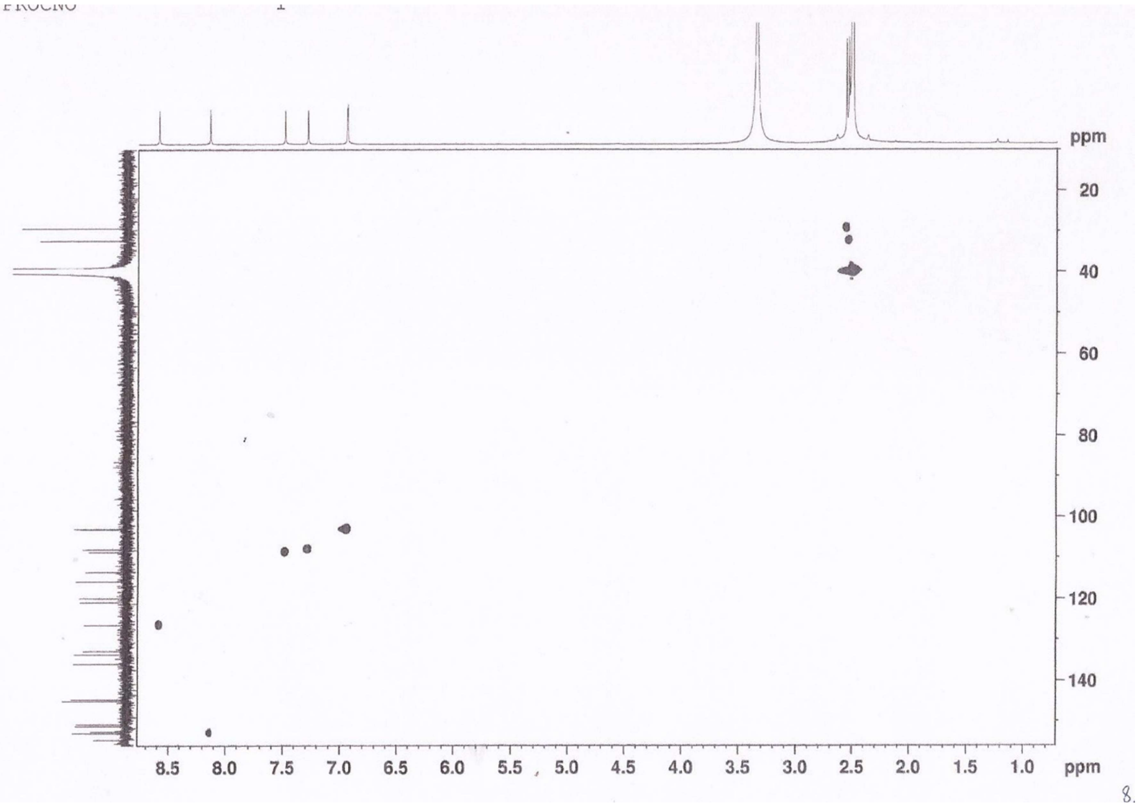

Figure S10. Expansion of the HSQC spectrum of SPF-3059-26 (**2**) (DMSO-d_6, 500 MHz).

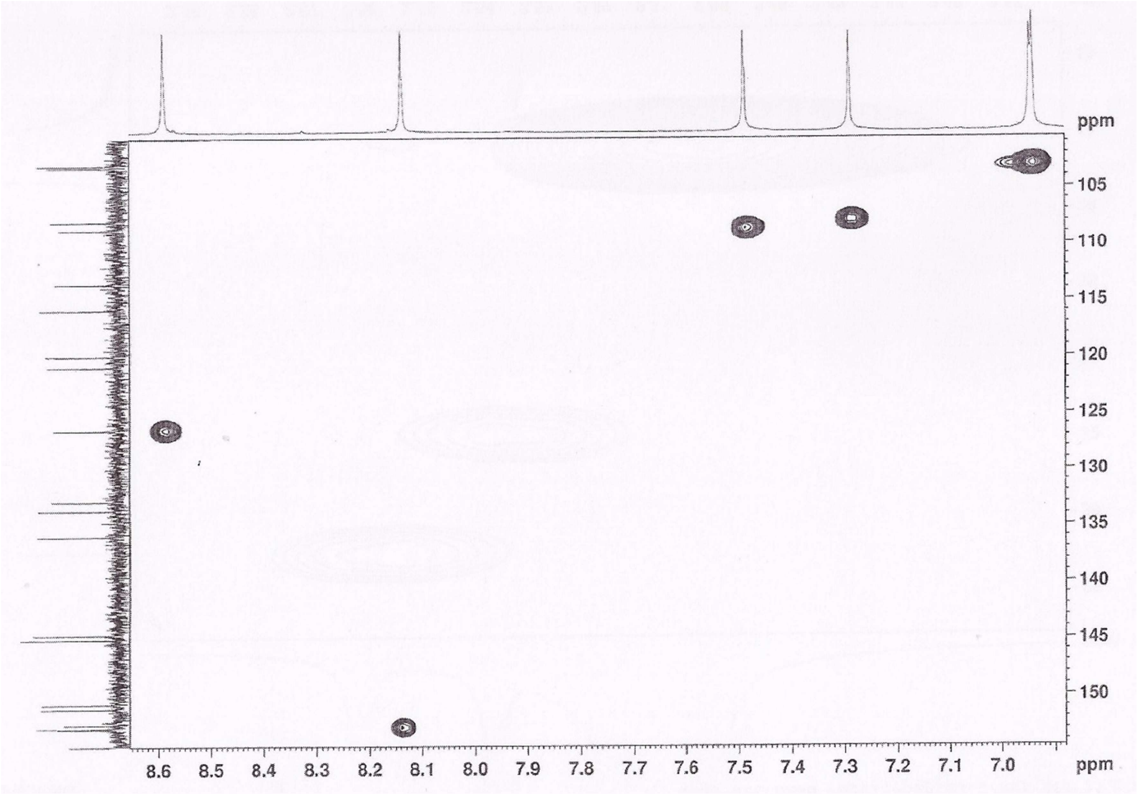

Figure S11. HMBC spectrum of SPF-3059-26 (**2**) (DMSO-d_6, 500 MHz).

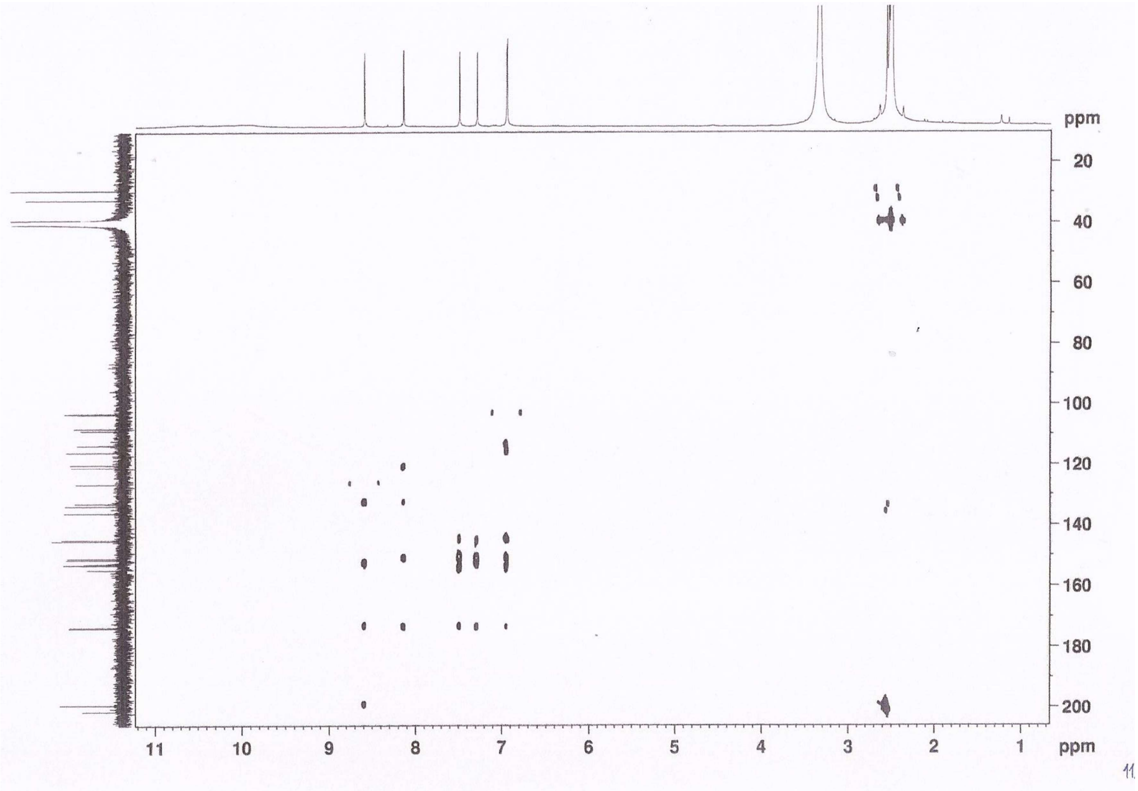

Figure S12. Expansion of the HMBC spectrum of SPF-3059-26 (**2**) (DMSO-d_6, 500 MHz).

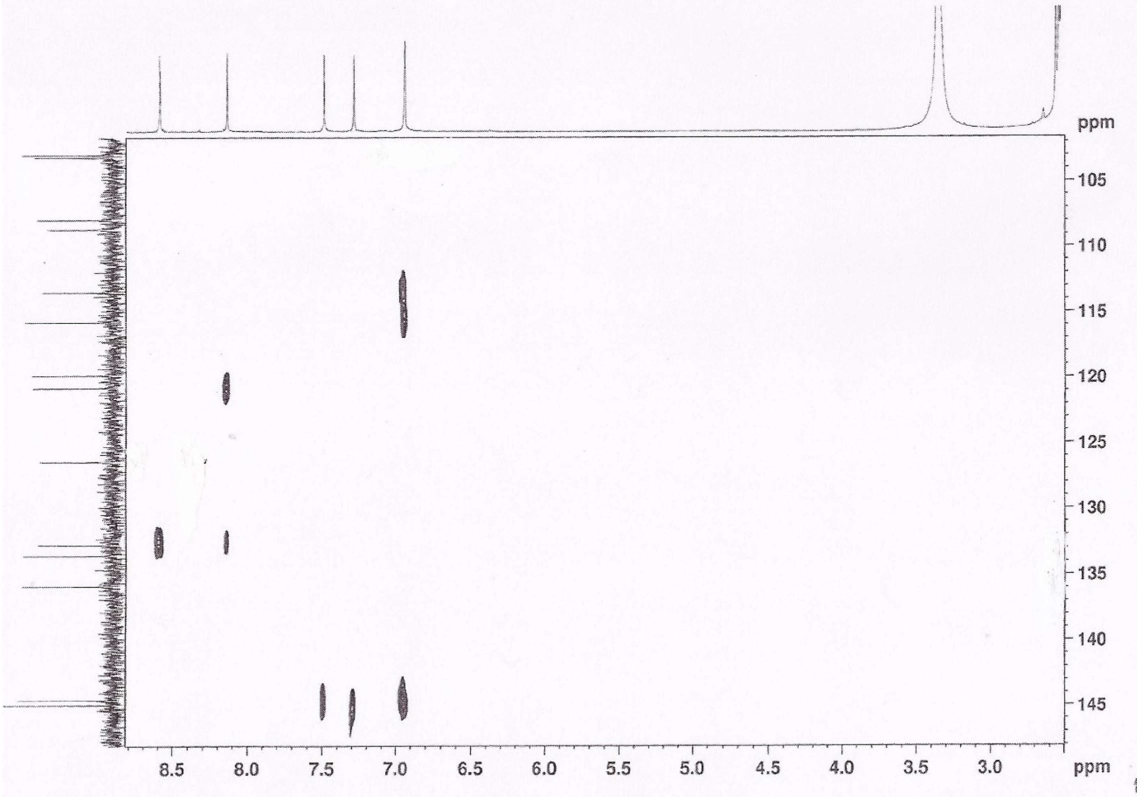

Figure S13. HRMS spectrum of erubescensoic acid (**1**)

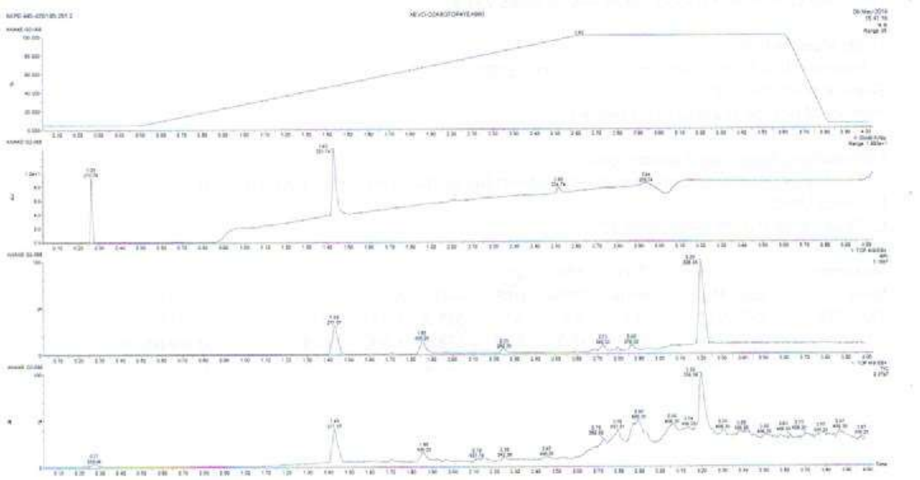

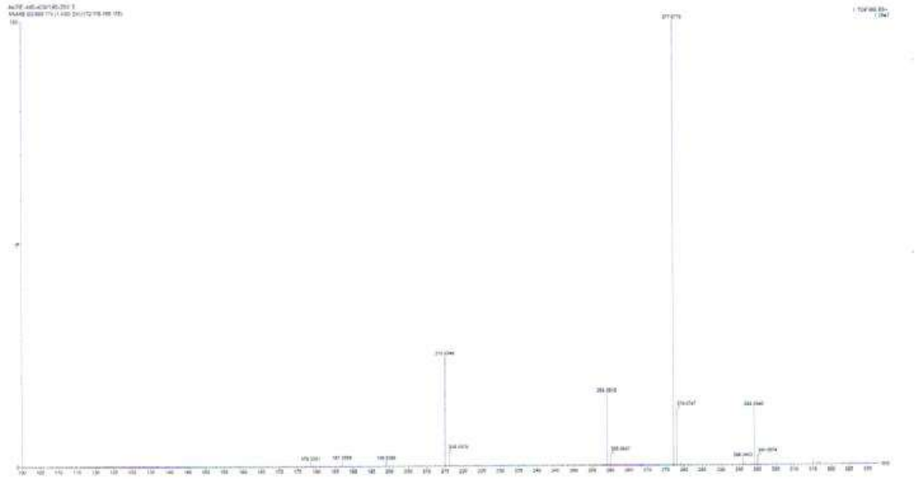

Elemental Composition Report: **AKPE 445-429/185-251.2**

Single Mass Analysis
Tolerance = 10.0 PPM / DBE: min = -1.5, max = 100.0
Element prediction: Off
Number of isotope peaks used for i-FIT = 3

Monoisotopic Mass, Even Electron Ions
299 formula(e) evaluated with 3 results within limits (up to 20 closest results for each mass)
Elements Used:
C: 10-18 H: 0-150 N: 0-30 O: 0-30

Minimum:				-1.5				
Maximum:		10.0	10.0	100.0				
Mass	Calc. Mass	mDa	PPM	DBE	i-FIT	Norm	Conf(%)	Formula
277.0719	277.0712	0.7	2.5	8.5	519.3	2.373	9.32	C14 H13 O6
	277.0726	-0.7	-2.5	13.5	518.6	1.680	18.64	C15 H9 N4 O2
	277.0699	2.0	7.2	14.5	517.2	0.328	72.04	C11 H5 N10

Figure S14. HRMS spectrum of SPF-3059-26 (**2**)

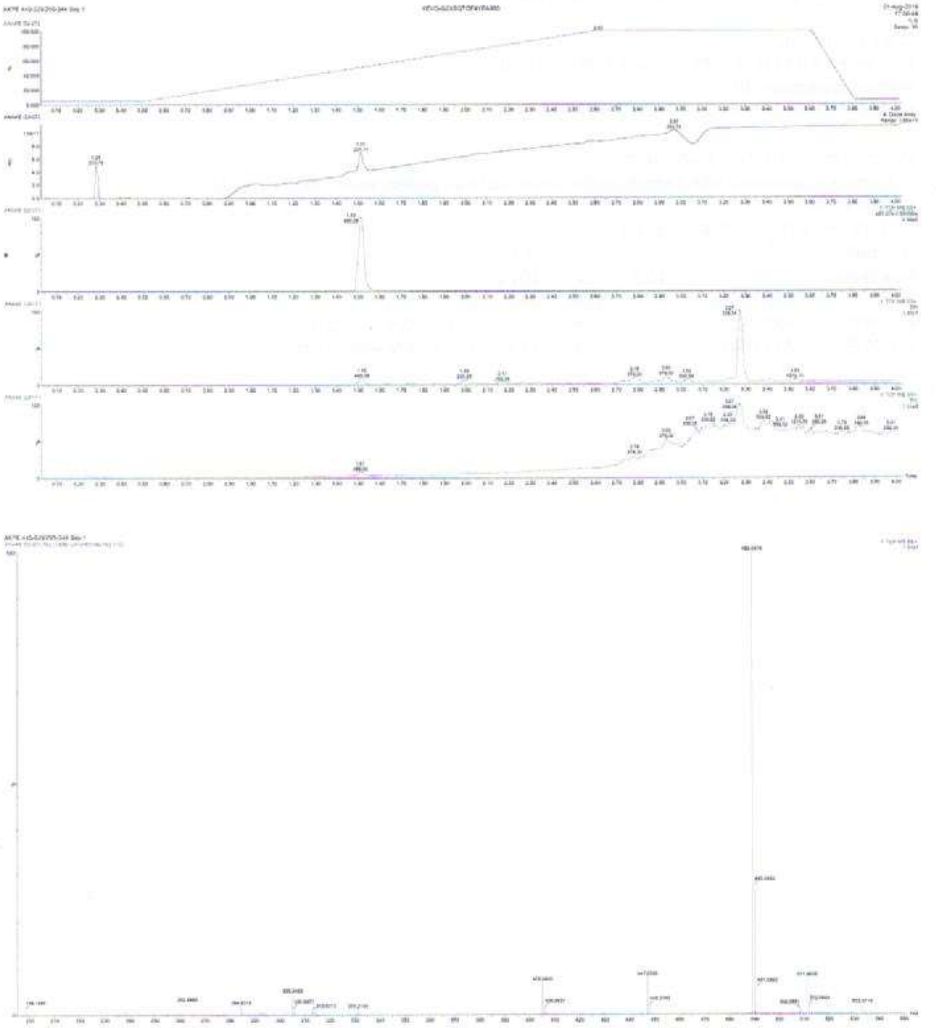

Elemental Composition Report: **AKPE 445-529/295-344 Sep 1**

Single Mass Analysis
Tolerance = 5.0 PPM / DBE: min = -1.5, max = 100.0
Element prediction: Off
Number of isotope peaks used for i-FIT = 3

Monoisotopic Mass, Even Electron Ions
31 formula(e) evaluated with 1 results within limits (up to 20 closest results for each mass)
Elements Used:
C: 26-26 H: 0-150 O: 0-30 Na: 1-1

Minimum:				-1.5		
Maximum:		10.0	5.0	100.0		
Mass	Calc. Mass	mDa	PPM	DBE	i-FIT	Formula
489.0818	489.0822	-0.4	-0.8	18.5	560.2	C26 H17 O10
511.0635	511.0641	-0.6	-1.2	18.5	199.3	C26 H16 O10 Na
